# Einfluss des Demenzgrades auf die motorischen Fähigkeiten von Senioren

**DOI:** 10.1007/s00391-021-01994-5

**Published:** 2021-11-11

**Authors:** Anneke Schumacher, Alexander Prinz, Kerstin Witte

**Affiliations:** Institut III, Bereich: Sportwissenschaft, Lehrstuhl Sport und Technik/Bewegungswissenschaft, Otto-von-Guericke-Universität Magdeburg, Zschokkestr. 32, 39104 Magdeburg, Deutschland

**Keywords:** Demenz, Kraftfähigkeiten, Gleichgewicht, Mobilität, ADL, Dementia, Strength ability, Balance, Mobility, Activities of daily living

## Abstract

**Hintergrund:**

Der progrediente Verlauf einer Demenzerkrankung äußert sich sowohl in den zunehmenden Beeinträchtigungen der kognitiven Funktionen als auch in den motorischen Fähigkeiten der Betroffenen und wirkt damit auf die Mobilität und Selbstständigkeit im Alltag aus. Die vorliegende Studie untersucht, ob und inwiefern der Schweregrad der Demenz ausgewählte motorische Leistungsfähigkeiten beeinflusst.

**Methodik:**

In die Studie wurden 32 SeniorenInnen mit einer leichten Demenz (Gruppe A; Altersdurchschnitt: 84,3 ± 6,1 Jahre) und 27 SeniorInnen mit einer mittelschweren Demenz (Gruppe B; Altersdurchschnitt: 85,6 ± 5,5 Jahre) eingeschlossen, deren Einstufung anhand des Mini-Mental-Status-Tests (MMST) erfolgte. Zum Einsatz kamen folgende motorische Testverfahren: Handdynamometer (Gesamtkraftfähigkeit), Fall-Stab-Test (motorische Reaktionsfähigkeit), Frailty and Injuries: Cooperative Studies of Intervention Techniques (FICSIT‑4; Gleichgewichtsfähigkeit), Timed-up-and-go-Test (TUG) und der modifizierte Chair-Rising-Test (CRT; beide analysieren die Mobilität und Transferleistung im Alltag).

**Ergebnisse:**

Gruppe A weist im Gegensatz zur Gruppe B eine signifikant bessere motorische Leistungsfähigkeit in allen motorischen Tests, außer dem Fall-Stab-Test, auf. Die größten Unterschiede liegen bei CRT (*p* = 0,000) und TUG (*p* = 0,011).

**Schlussfolgerung:**

Bezüglich der Handkraft, der Gleichgewichtsfähigkeit und der Mobilität weisen Menschen mit einem höheren Demenzgrad eine geringere Leistungsfähigkeit auf. In nachfolgenden Studien zur Auswirkung der Demenzerkrankung auf unterschiedliche Fähigkeiten und Fertigkeiten sollte deshalb stets eine Einteilung der Demenzgrade erfolgen. Zudem ist zu empfehlen, dass sich Interventionsprogramme auf die allgemeine Kraft- sowie Gleichgewichtsfähigkeit fokussieren sollten.

**Zusatzmaterial online:**

Zusätzliche Informationen sind in der Online-Version dieses Artikels (10.1007/s00391-021-01994-5) enthalten.

## Abstract (en)

**Background:**

The progressive course of dementia is not only manifested in the increasing impairment of cognitive functions but also in the motor skills of those affected and also impairs their mobility and independence in coping with everyday tasks. The present study investigated whether and to what extent there is a difference in selected skills of motor performance depending on the stages of dementia.

**Material and methods:**

This study included 32 senior citizens with mild dementia (group A; 84.3 ± 6.1 years) and 27 senior citizens with moderate dementia (group B; 85.6 ± 5.5 years), whose classification was based on the mini-mental state examination (MMSE). The following motor tests were used: the hand dynamometer test (general strength), the drop bar test (motor reaction ability), frailty and injuries: cooperative studies of intervention techniques (FICSIT‑4; balance), the timed up-and-go test (TUG) and the modified chair rising test (CRT; both assess mobility and transfer performances in everyday life).

**Results:**

Group A, in contrast to group B, showed significantly better motor performance in all motor tests except the drop bar test. The largest differences were found in the CRT (*p* = 0.000) and TUG (*p* = 0.011).

**Conclusion:**

In terms of overall strength, balance and mobility, people with more advanced dementia showed lower performance. Therefore, subsequent studies investigating the impact of dementia on motor skills should always include a classification of dementia levels. Intervention programs should contain physical activities that focus on increasing general strength as well as balance.

Dem Krankheitsbild der Demenz liegen strukturelle und funktionelle Veränderungen des Gehirns zugrunde, deren Prozesse und Symptome den altersbedingten Leistungsabfall hinsichtlich der kognitiven Funktion und spezifisch der Gedächtnisleistung weitaus übersteigen. Neben der Minderung der kognitiven Leistungsfähigkeit stellt sich zudem ein Abbau der motorischen Fähigkeiten ein, der sich auf die Mobilität und die Selbstständigkeit des Betroffenen auswirkt. In diesem Beitrag soll die motorische Leistungsfähigkeit in Abhängigkeit vom Schweregrad der Demenz untersucht werden.

Bereits bei den natürlichen Degenerationen der Gehirnstrukturen gesunder älterer Menschen zeigen sich Atrophien in allen kortikalen sowie subkortikalen Regionen, die sich insbesondere im präfrontalen und temporalen Kortex bemerkbar machen. Dadurch ist ein Leistungsabfall der Exekutivfunktionen, der Aufmerksamkeit, des episodischen Gedächtnisses und des Arbeitsgedächtnisses zu verzeichnen [9, 14]. Zudem können bei dem Krankheitsbild der Demenz Einbußen der räumlichen und zeitlichen Orientierung entstehen, die sich in Kombination mit den zuvor genannten alters- und demenzbedingten kognitiven Defiziten einerseits anhand von Einschränkungen der funktionellen Alltagsleistungen („instrumental activities of daily living“) der Betroffenen zeigen und andererseits mit dem Verlust von motorischen Basisleistungen einhergehen [13]. Dies hat Auswirkungen auf die Selbstständigkeit von Demenzpatienten und die Bewältigung alltäglicher Aufgaben. Die motorischen Fertigkeiten des Gehens, der Transferbewegungen (z. B. Aufstehen von einem Stuhl) und der Gleichgewichtsfähigkeit sind in der Planung und Bewegungskontrolle beeinträchtigt [13, 14].

Der fortschreitende Verlauf der Demenz geht mit zunehmenden kognitiven Beeinträchtigungen einher, sodass das Krankheitsbild grundlegend in 3 Schweregrade unterteilt wird: die leichte, die mittelschwere und die schwere Demenz. Dabei nimmt das Clinical Dementia Rating (CDR) von Hughes et al. [5] die klinische Symptomatik umfassend auf und dient bis heute noch weltweit zur Einschätzung dieser Schweregrade. Das CDR bietet eine klare Trennung in der Entwicklung der Demenz von CDR 0 (gesund), CDR 0,5 (sehr leicht), CDR 1 (leicht), CDR 2 (mittelschwer) und CDR 3 (schwer). In Forschungsstudien wird meist aus ökonomischen Gründen vorranging der Mini-Mental-Status-Test (MMST), der eigentlich ein Bestandteil des CDR darstellt, als eigenständiges Screeningverfahren zur Ermittlung des Schweregrades eingesetzt. Der Punkte-Score des MMST wird mit dem CDR-Score gleichgesetzt [10].

Die Einteilung dieser Demenzgerade findet in der aktuellen Forschungslage bezüglich der Untersuchungen kognitiver Beeinträchtigungen vermehrt Anwendung. Es liegen allerdings wenige Studien vor, die einen Zusammenhang zwischen der Schwere der kognitiven Beeinträchtigung einer Demenzerkrankung und der motorischen Leistungsfähigkeit darstellen. Eine Querschnittsstudie von Zidan et al. [15] untersuchte die motorischen und funktionalen Veränderungen in Bezug auf die Stadien der fortschreitenden Alzheimer-Demenz (AD) gegenüber der Leistungsfähigkeit von gesunden älteren Menschen. Signifikante Unterschiede stellten sich v. a. in einer Vielzahl von Tests zu kognitiven und motorischen Funktionen sowie zur Aktivität im Alltag (ADL; „activities of daily living“) im Gruppenvergleich der Testpersonen der CDR 1 und CDR 2 der AD heraus. Tangen et al. [12] bestätigten ebenfalls einen Zusammenhang zwischen der Gleichgewichtsfähigkeit und den kognitiven Fähigkeiten bei Patienten mit kognitiven Beeinträchtigungen. Testergebnisse von Personen mit einer subjektiven kognitiven Beeinträchtigung (SCI; „subjective cognitive impairment“), milden kognitiven Beeinträchtigung (MCI; „mild cognitive impairment“) sowie einer leichten und mittelschweren AD wurden verglichen, deren Einteilung anhand vorliegender klinischer Diagnosen stattfand. Zur Analyse der kognitiven Leistungsfähigkeit kamen u. a. Teile des Consortium to Establish a Registry for Alzheimer’s Disease (CERAD-Plus) zum Einsatz. Zwischen den Ergebnissen des Balance Evaluation Systems Test (BESTest) und dem Schweregrad der kognitiven Beeinträchtigung der Probanden stellte sich ein Zusammenhang heraus: Je ausgeprägter und somit fortgeschrittener die kognitive Beeinträchtigung ist, desto schlechter ist die Gleichgewichtsfähigkeit.

Der aktuelle Forschungsstand belegt zwar die verminderte motorische Leistungsfähigkeit von Demenzpatienten, aber nur unzureichend die Abhängigkeit der motorischen Leistungsfähigkeit vom Schweregrad der Demenzerkrankung. Das Ziel dieser Studie ist es herauszufinden, ob und inwieweit der Schweregrad einer Demenz, bestimmt durch den MMST, die Motorik hinsichtlich verschiedener Kraftfähigkeiten, der Gleichgewichtsfähigkeit, der Reaktionsfähigkeit sowie der Mobilität beeinflusst.

## Methodik

### Stichprobe

Insgesamt nahmen 59 Seniorinnen und Senioren im Alter zwischen 69 und 94 Jahren aus verschiedenen Pflege- und Tageseinrichtungen teil, unter denen 32 Probanden eine leichte Demenz (Gruppe A) und 27 Probanden eine mittelschwere Demenz (Gruppe B) aufweisen. Die Einstufung der Schwergrade erfolgte durch die erzielten Punkte im MMST, da die klinischen Daten der Probanden nicht vollständig vorlagen. Eine Vorauswahl der Studienteilnehmer erfolgte aus organisatorischen und coronabedingten Gründen durch das Pflegepersonal der verschiedenen Heim- und Tageseinrichtungen, unter Berücksichtigung der gesetzten Ein- bzw. Ausschlusskriterien. Aufgrund der geringen Stichprobengröße der Senioren mit einer schweren Demenz fokussierte sich diese Untersuchung lediglich auf die Gruppen der leichten und mittelschweren Demenz (Tab. 1), deren Einverständniserklärungen bzw. die der bevollmächtigten Angehörigen vorlagen. Die Stichprobencharakteristik ist der Tab. 1 zu entnehmen. Bezüglich der „Sturzhäufigkeit“ und der „Gangunsicherheit“ wurden die dokumentierten Stürze der letzten 6 Monate sowie die subjektiven Einschätzungen der Pfleger bzw. der Angehörigen der Probanden zum eigenständigen Gang anhand einer 5‑stufigen Likert-Skala notiert. Beide Stichproben unterscheiden sich anhand ihrer Merkmalsausprägungen, abgesehen vom MMST-Wert, nicht signifikant voneinander.

**Table 1:** 

	Demenzgruppe		χ^2^-Test
Merkmal	Gruppe A (leichte Demenz) (*n* = 32) MW ± SD	Gruppe B (mittelschwere Demenz) (*n* = 27) MW ± SD	Signifikanz *p*
MMST-Wert (Punkte)	23,7 ± 2,1	13,6 ± 4,4	0,000
Geschlecht (m/w)	6/26	6/21	0,741
Alter (Jahre)	84,3 ± 6,1	85,6 ± 5,5	0,094
BMI (kg/m^2^)	28,4 ± 5,4	26,6 ± 4,6	0,439
Jahre, Heimaufenthalt	1,9 ± 2,7	2,6 ± 2,6	0,240
Sturzhäufigkeit (6 Monate)	0,3 ± 0,6	0,6 ± 1,1	0,635
Gangunsicherheit	2,3 ± 1,3	2,6 ± 1,3	0,704
Art der Demenz (%)	59,4 % (nicht genau definiert); 37,5 % (AD); 3,1 % (vaskulär)	63,0 % (nicht genau definiert); 25,9 % (AD); 11,1 % (vaskulär)	0,365

*MW* Mittelwert, *SD* Standardabweichung

### Motorische Testverfahren

Zur Untersuchung der motorischen Leistungsfähigkeit der Seniorinnen und Senioren kamen verschiedene standardisierte Tests in immer derselben Reihenfolge zum Einsatz, die die Gesamtkraftfähigkeit und Reaktionsgeschwindigkeit der oberen Extremitäten, die Gleichgewichtsfähigkeit sowie die Kraftfähigkeit der unteren Extremitäten erfassen (Zusatzmaterial online). Die Testung mittels Handdynamometer, der Fall-Stab-Test, der FICSIT‑4, der Timed-up-and-go-Test *(TUG)* sowie der Chair-Rising-Test *(CRT)* eignen sich als motorische Testverfahren für Demenzpatienten und wurden bereits in vorherigen Studien verwendet [3, 6, 8]. Die Messungen aller Probanden erfolgte in allen Einrichtungen durch dieselbe Testleitung.

### Statistische Auswertung

Die erhobenen Daten der verschiedenen motorischen Testverfahren wurden in SPSS (Version 26) ausgewertet. Der Vergleich der Gruppenergebnisse erfolgte nach Prüfung auf Normalverteilung der Daten anhand des *t*-Tests für unabhängige Stichproben sowie des Mann-Whitney-U-Tests der nichtnormalverteilten Ergebnisse. Des Weiteren wurde der Chair-Rising-Test hinsichtlich des Faktors der Hilfestellung sowie des Interaktionseffekts zwischen dem Demenzgrad und der Hilfestellung durch die zweifaktorielle Varianzanalyse untersucht.

Im ersten Schritt folgte hierbei eine Gruppenaufteilung der Teilnehmerdaten in die Gruppe C (mit Hilfe; *n* = 22) und Gruppe D (ohne Hilfe; *n* = 32), ohne den Faktor des Demenzgrades zu berücksichtigen, deren Mediane mittels Mann-Whitney-U-Tests überprüft wurden. Im zweiten Schritt wurde der anhand der Hilfestellung aufgeteilte Datensatz (Gruppen C und D) unter Hinzunahme der Gruppenvariable des Demenzgrades auf signifikante Unterschiede getestet, um zu sehen, ob der Demenzgrad einen Einfluss auf die motorische Leistung hat, wenn eine Hilfestellung benötigt wird oder nicht.

Signifikante Unterschiede beider statistischen Auswerteverfahren wurden aufgrund der in etwa gleichgroßen Stichproben durch die Effektstärke des Korrelationskoeffizienten (r) von Pearson berechnet und dahingehend eine Beurteilung der Effektgröße anhand der Einteilung von Cohen vorgenommen. Ein schwacher Effekt liegt ab r ≥ 0,10, ein mittlerer Effekt ab r ≥ 0,30 und ein starker Effekt ab r ≥ 0,50 vor [4].

## Ergebnisse

Die Tab. 2 stellt die Mittelwerte (MW) und die Streuung (SD; Standardabweichung) der motorischen Testverfahren beider Gruppen in Abhängigkeit zum Demenzgrad dar und zeigt neben der Prüfung der Mittelwerte auf Signifikanzen ebenso die Effektstärke r.

**Table 2:** 

Variable	Gruppe	*n*	MW	SD	Signifikanz *p*	Effektstärke r
Handdynamometer, rechte Hand (N) ↑	A	32	167,8	±61,2	0,030^M^	0,28
	B	27	137,5	±65,8		
Handdynamometer, linke Hand (N) ↑	A	32	144,4	±51,7	0,015^M^	0,32
	B	27	113,3	±55,1		
Fall-Stab-Test (cm) ↓	A	31	26,6	±12,28	0,909	–
	B	26	27	±13,5		
FICSIT‑4 (Gesamtpunktzahl) ↑	A	32	16,7	±7,3	0,026^M^	0,29
	B	27	11,7	±9,1		
Timed-up-and-go-Test (s) ↓	A	31	19,6	±8,41	0,011^M^	0,34
	B	26	26,6	±10,6		
Chair-Rising-Test (s) ↓ *Demenzgrad*	A	31	16,1	±6,54	0,000^M^	0,52
	B	23	23,5	±10,9		
Chair-Rising-Test (s) ↓ *Mit und ohne Hilfestellung*	C	22	25,2	±10,8	0,000^M^	0,58
	D	32	15,1	±5,18		

↑ und ↓ zeigen an, ob ein hohes oder niedriges Testergebnis als optimal einzuschätzen ist

*MW* Mittelwert, *SD* Standardabweichung, *N* Newton, *M* Testung mittels Mann-Whitney-U-Test, *Gruppe A* leichte Demenz, *Gruppe B* mittelschwere Demenz, *Gruppe C* mit Hilfestellung, *Gruppe D* ohne Hilfestellung

Von den getesteten 59 Probanden liegen Abweichungen der Stichprobengröße *n* im Fall-Stab-Test sowie TUG und CRT vor, da diese aufgrund von körperlichen Beschwerden zum Messzeitpunkt nicht von allen Probanden durchführbar waren.

Im Allgemeinen konnten die Probanden der Gruppe A in allen Tests besser abschneiden als die Probanden der Gruppe B. Die Ergebnisse weisen signifikante Unterschiede hinsichtlich der Handkraft sowie der Kraft der unteren Extremitäten und der Gleichgewichtsfähigkeit mit leichten bis starken Effekten auf, die durch den Demenzgrad der Testpersonen begründet werden. Lediglich die Leistung der Reaktionsfähigkeit, die durch den Fall-Stab-Test gemessen wurde, konnte nicht signifikant bestätigt werden.

Da die Durchführung des CRT nicht von allen Senioren ohne Hilfestellung absolviert werden konnte, ist es schwierig, eine Aussage zur motorischen Leistung allein anhand der gemessenen Zeit zu treffen. Deshalb wurde protokolliert, wie viele TeilnehmerInnen eine Hilfestellung und wie viele keine Hilfe benötigten. Die Verteilung dieser gekreuzten Daten der Vierfeldertafel (Tab. 3) weist unter Anwendung des χ^2^-Tests, mit keinen erwarteten Zellhäufigkeiten kleiner 5, einen statistisch signifikanten Zusammenhang (χ^2^ (1) = 4,13; *p* = 0,042) zwischen den Gruppenmerkmalen des Demenzgrades und der Hilfestellung im CRT auf. Hier liegt eine mittlere Effektstärke ϕ = 0,277 durch die Einteilung der Effektstärkenmaße nach Cohen vor. Patienten mit einer mittelschweren Demenz brauchen öfter eine Hilfestellung im CRT als Patienten mit einem leichten Schweregrad der Demenz. Somit lässt sich einschätzen, dass Menschen mit einer mittelschweren Demenz bei alltäglichen Aktivitäten stärker eingeschränkt sind als Menschen, die einen leichten Demenzgrad besitzen. Zusätzlich zeigte sich ein signifikanter Unterschied zwischen den Gruppen C und D (Hilfestellung oder keine Hilfestellung) im CRT hinsichtlich der Dauer der Testausführung mit einem starken Effekt (Tab. 2). Hier konnte allerdings kein Interaktionseffekt (F = 0,338; *p* = 0,564; η^2^ = 0,007) zwischen dem Demenzgrad und der Hilfestellung bezüglich der Kraftfähigkeit im CRT nachgewiesen werden.

**Table 3:** 

Demenzgrad	Hilfestellung	
	Gruppe C (ja)	Gruppe D (nein)
*Gruppe A (leichte Demenz)*	9	22
*Gruppe B (mittelschwere Demenz)*	13	10

## Diskussion

Ziel dieser Studie ist es, die Unterschiede der motorischen Leistungsfähigkeit von Demenzpatienten in Abhängigkeit vom Schweregrad der Erkrankung festzustellen. Dabei wurde nicht die Art der Demenz berücksichtigt. Zum Einsatz kamen zahlreiche motorische Testverfahren, die die Gesamtkraftfähigkeit und Reaktionsfähigkeit der oberen Extremitäten, die Gleichgewichtsfähigkeit sowie die Kraftfähigkeit der unteren Extremitäten analysierten.

Bislang sind in der Literatur wenige Untersuchungen zu finden, die sich mit der motorischen Leistungsfähigkeit von Demenzpatienten in Abhängigkeit vom individuellen Schweregrad der Erkrankungen befassen. Wie die vorliegenden Ergebnisse dieser Studie zeigen, besteht ein deutlicher Unterschied in der motorischen Leistung zwischen Patienten mit einer leichten Demenzerkrankung (Gruppe A) und Patienten mit einem mittelschweren Demenzgrad (Gruppe B). Insbesondere im TUG und im CRT treten mittlere und hohe Effekte zwischen den Gruppen auf, die den Verlust von motorischen Basisleistungen widerspiegeln, da in diesen Tests v. a. die motorischen Schlüsselqualifikationen des Gehens sowie der Transferleistung (Aufstehen von einem Stuhl) erfasst werden. Die mit der Demenzerkrankung voranschreitenden kognitiven Beeinträchtigungen wirken sich demnach auch auf die motorischen Fähigkeiten aus. Somit werden die Ergebnisse der Untersuchung von Zidan et al. [15] bestätigt, die einen Leistungsabfall der motorischen Fähigkeiten bei einer fortschreitenden AD (CDR1–CDR3) gegenüber gesunden älteren Menschen nachweisen. Auch hier zeigen sich u. a. hochsignifikante Unterschiede im TUG sowie im Sit-to-stand-Test, die den Gang und die Transferleistung des Aufstehens erfassen. Demzufolge verfügen Patienten mit einem leichten Demenzgrad generell über eine bessere motorische Leistungsfähigkeit als Patienten, bei denen bereits ein mittelschwerer Demenzgrad vorliegt. Umso wichtiger ist es, die Erkrankung frühzeitig zu diagnostizieren und ihren Verlauf mit geeigneten Interventionsmaßnahmen, solange es geht, stagnieren zu lassen.

Des Weiteren tritt ein starker Effekt im Gruppenvergleich hinsichtlich der Hilfestellung im CRT auf. Probanden der Gruppe C, die Hilfe bei der Bewältigung der Testaufgabe benötigen, weisen eine signifikant geringere motorische Leistungsfähigkeit auf, als Probanden der Gruppe D, die keine Hilfestellung jeglicher Form brauchen. Diesbezüglich wird durch den χ^2^-Test aufgezeigt, dass ein signifikanter Unterschied zwischen den Gruppenmerkmalen Demenzgrad und Hilfestellung besteht. Deutlich mehr Teilnehmer mit einer mittelschweren Demenz sind auf die Hilfestellung angewiesen als Teilnehmer mit einer leichten Demenz. Demzufolge kann darauf geschlossen werden, dass Patienten mit einem fortgeschrittenen Demenzverlauf eine eingeschränktere motorische Leistungsfähigkeit besitzen, somit hilfebedürftiger sind und auf Unterstützung im Alltag sowie bei der Bewältigung von funktionellen Alltagsleistungen angewiesen sind.

Hinzu kommt, dass die Gesamtkraftfähigkeit, bestimmt durch ein Handdynamometer, der Gruppe B sichtbar schlechter ist als die der Gruppe A. Patienten mit einer mittelschweren Demenz, deren Griffkraft signifikant unter den Werten der Patienten einer leichten Demenz liegen, verfügen zudem über eine geringere isometrische Kraftfähigkeit bezüglich der Muskulatur der unteren Extremitäten. Der Zusammenhang der Leistungsfähigkeit dieser Muskelgruppen ist durch Lauretani et al. [7] belegt und wird mit den Ergebnissen des FICSIT‑4, des TUG und des CRT dieser Untersuchung gestützt.

Nur bei der motorischen Reaktionsfähigkeit macht sich eine Differenzierung des Schweregrades der Demenz nicht bemerkbar und zeigt keinen signifikanten Unterschied in den Ergebnissen des Fall-Stab-Tests. Dies könnte darauf zurückzuführen sein, dass das gewählte motorische Testverfahren möglicherweise nicht sensibel genug ist und die motorische Reaktionsfähigkeit unter Anwendung anderer spezifischer Tests genauer analysiert werden könnte. Die Studienlage ist diesbezüglich noch unzureichend. In der Literatur gibt es zwar Hinweise darauf, dass die Reaktionsfähigkeit von Betroffenen im Gegensatz zu gesunden Senioren und MCI-Patienten vermindert ist, allerdings liegen hier keine empirischen Daten unter dem Aspekt des Einflusses des Schweregrades der Demenz vor.

Eine Erklärung für die große Streuung der erhobenen Messergebnisse im Fall-Stab-Test ist, dass spezifische Reizstimuli auch komplexe kognitive Verarbeitungsprozesse benötigen, welche bei Demenzpatienten individuell verlaufend eingeschränkt sind. Dabei ist die Reaktionszeit laut Andriuta et al. [1] von kritischen Prozessen, dem sog. kombinierten Index, abhängig. Dieser setzt sich aus (1) Wahrnehmungsprozessen über relevante und irrelevante Reize, (2) der Entscheidung, Reaktionen auszulösen, (3) einem motorischen Verfahren über verschiedene motorische Kontrollsysteme bis zur Ausführung der Reaktion und (4) einem zentralen Aufmerksamkeitsprozess (anhaltende Wachsamkeit) zusammen. Jeder dieser Prozesse kann die Reaktionszeit beeinflussen, sodass selbst die Messung der einfachen Reaktionszeit eine Analyse der intraindividuellen Variabilität über eine Testbatterie erfordert. Die intraindividuelle Reaktionszeitvariabilität, ein Verhaltensmarker für die neurologische Integrität von kognitiven Erkrankungen, nimmt bei MCI- und Demenzpatienten zu und spiegelt sich insbesondere auf der Ebene der Reaktionszeit von einfachen Reizen mit einer geringen Verarbeitungslast wider [1, 11]. Baddeley et al. [2] bestätigen zudem, dass die Aufmerksamkeitskontrolle der exekutiven Funktion bereits im frühen AD-Stadium abnimmt und sich insbesondere im Bereich der geteilten Aufmerksamkeit und der Selektion von Störreizen äußert.

Da in der Literatur dennoch bisher wenig über die Wirkmechanismen der aufmerksamkeitsbedingten Verlangsamung der Reaktion in den verschiedenen Schweregraden der Demenz vorliegt, sollten die unterschiedlichen Stadien der Erkrankung bezüglich ihrer Reaktionsfähigkeit in den unterschiedlichen Exekutivprozessen getrennt analysiert werden [2, 11].

### Limitationen

Folgende Kriterien hinsichtlich des Studiendesigns schränken die Aussagekraft der Untersuchungsergebnisse ein:

- geringe Stichprobengröße aus 2 Seniorenheimen,
- keine Einteilung der Schweregrade mittels umfassender klinischer Diagnostik,
- Randomisierung der Stichprobe erfolgte nach einer Vorauswahl des Pflegepersonals durch den MMST,
- kein Vergleich zwischen gesunden Senioren (Kontrollgruppe) und allen Schweregraden der Demenz,
- Beschreibung der Demenzart.

## Ausblick

Um den dargelegten motorischen Defiziten in den Bereichen von Kraft und Gleichgewicht entgegenzuwirken bzw. diesem Leistungsabbau im Verlauf einer Demenzerkrankung gezielt aufzuhalten, ist es nicht nur wichtig, dass geeignete Interventionsmaßnahmen zum Einsatz kommen, sondern ebenso, dass diese Maßnahmen an den individuellen Leistungsstand des Patienten angepasst werden. Zukünftige Trainingsinterventionen sind so zu konzipieren, dass ein spezifisches Kraft- oder Gleichgewichtstraining in Abhängigkeit vom Schweregrad der Demenz zum Einsatz kommt und demnach eine Unterteilung der individuellen physiologischen, kognitiven und motorischen Leistungsfähigkeiten von Demenzpatienten beinhaltet.

## Fazit für die Praxis

- Ein höherer Demenzgrad bedeutet einerseits eine stärkere Beeinträchtigung der kognitiven Fähigkeiten, aber andererseits auch eine reduzierte motorische Leistungsfähigkeit.
- Zukünftige Interventionsstudien und Therapien in Bezug auf die Verbesserung der Mobilität sollten den Schweregrad der Demenz berücksichtigen.

## Supplementary Information




