# Supplementary material for: Influence of the dementia stage on motor skills in senior citizens
Source: Z Gerontol Geriatr. 2021 Nov 11;56(1):29–34. [Article in German] doi: 10.1007/s00391-021-01994-5 (PMC9877064; doi:10.1007/s00391-021-01994-5)
Supplement: Supplementary file 1 [file 391_2021_1994_MOESM1_ESM.docx]

**Supplement 1. Motorische Testverfahren**

Zur Untersuchung der motorischen Leistungsfähigkeit der Seniorinnen und Senioren kamen verschiedene standardisierte Tests zum Einsatz, die die Gesamtkraftfähigkeit und Reaktionsgeschwindigkeit der oberen Extremitäten, die Gleichgewichtsfähigkeit sowie die Kraftfähigkeit der unteren Extremitäten erfassen. Diese Testverfahren sind auch für Demenzpatienten geeignet und wurden in vorherigen Studien verwendet [1,4,6].

Zur Einschätzung der Griffkraft wurde ein *Handdynamometer* verwendet. Der beste Versuch der linken und rechten Hand (in Newton) aus drei Durchgängen wurde ausgewertet [3].

Mithilfe des *Fall-Stab-Tests* (nach Fetz & Kornexl, entwickelt 1978) wird die motorische Reaktionsfähigkeit erfasst [2]. Hier zählte das beste Ergebnis (in cm) der präferierten Hand des Teilnehmers nach drei Durchgängen.

Mit dem *Frailty and Injuries: Cooperative Studies of Intervention Techniques (FICSIT)* kann die Gleichgewichtsfähigkeit von älteren Menschen untersucht werden. Der FICSIT-4 ist ein angepasstes Testverfahren, der vier verschiedene Stände testet: (1) der bipedale Stand mit geschlossenen Füßen, (2) der Semi-Tandem-Stand, (3) der Tandem-Stand sowie (4) der monopedale Stand auf dem präferierten Bein [7,9,10].

Zur Beurteilung der Mobilität und des Sturzrisikos von älteren Menschen wurde der *Timed-Up-and-Go-Test (TUG)* angewendet. Er analysiert die Transferleistung vom Aufstehen und Gehen. Hilfsmittel, wie z. B. Rollatoren oder Gehstöcke sind erlaubt. Die gestoppte Zeit des Durchgangs kann mittels Auswertebogen mit zeitlicher Einteilung Aufschluss über die Mobilitätseinschränkung im Alltag der Probanden geben [8].

Der modifizierte *Chair-Rising-Test (CRT)* dient der Ermittlung der muskulären Leistungsfähigkeit der unteren Extremitäten. Hier werden zwei Arten der Hilfestellung für die Versuchsperson geboten: Sie können sich entweder mit den Armen von den Stuhllehnen abstützen oder, für Personen mit einem schlechteren physischen Zustand, zusätzlich Hilfe vom Testleiter beim Aufstehen erhalten [5].

**Literatur**

1. Burton E, Cavalheri V, Adams R et al. (2015) Effectiveness of exercise programs to reduce falls in older people with dementia living in the community: a systematic review and meta-analysis. Clin Interv Aging 10: 421–434

2. Fetz F, Kornexl E (1993) Sportmotorische Tests, 3. Aufl. ÖBV Pädagog. Verl., Wien

3. Köppel M, Hamacher D (2019) Kräftigung wider das Altern. EHK 68: 116–123

4. Lai CH, Chen HC, Liou TH et al. (2019) Exercise Interventions for Individuals With Neurological Disorders: A Systematic Review of Systematic Reviews. Am J Phys Med Rehabil 98: 921–930

5. Le Berre M, Apap D, Babcock J et al. (2016) The psychometric properties of a modified sit-to-stand test with use of the upper extremities in institutionalized older adults. percept mot skills 123: 138–152

6. Lin YP, Yang YH, Hsiao SF (2019) Physical activity, muscle strength, and functional fitness. Topics Geriatr Rehabil 35: 280–288

7. Ory MG, Schechtman KB, Miller JP et al. (1993) Frailty and injuries in later life: the FICSIT trials. J Am Geriatr Soc 41: 283–296

8. Podsiadlo D, Richardson S (1991) The timed "Up & Go": a test of basic functional mobility for frail elderly persons. J Am Geriatr Soc 39: 142–148

9. Rossiter-Fornoff JE, Wolf SL, Wolfson LI et al. (1995) A cross-sectional validation study of the FICSIT common data base static balance measures. Frailty and Injuries: Cooperative Studies of Intervention Techniques. J Gerontol A Biol Sci Med Sci 50: M291-7

10. Thomas JC, Odonkor C, Griffith L et al. (2014) Reconceptualizing balance: attributes associated with balance performance. Exp Gerontol 57: 218–223
